# An Optical Reusable 2D Radiochromic Gel-Based System for Ionising Radiation Measurements in Radiotherapy

**DOI:** 10.3390/molecules29112558

**Published:** 2024-05-29

**Authors:** Marek Kozicki, Piotr Maras

**Affiliations:** 1 Department of Mechanical Engineering, Informatics and Chemistry of Polymer Materials, Faculty of Materials Technologies and Textile Design, Lodz University of Technology, Żeromskiego 116, 90-543 Lodz, Poland; 2 GeVero Co., 90-980 Lodz, Poland; 3 Department of Radiotherapy Planning, Copernicus Hospital, Pabianicka 62, 93-513 Lodz, Poland; piotr.maras@wp.pl

**Keywords:** NBT–Pluronic F-127, radiotherapy dosimetry, quality assurance, 2D dosimetry, polyGeVero-CT

## Abstract

This work describes the development of a reusable 2D detector based on radiochromic reaction for radiotherapy dosimetric measurements. It consists of a radiochromic gel dosimeter in a cuboidal plastic container, scanning with a flatbed scanner, and data processing using a dedicated software package. This tool is assessed using the example of the application of the coincidence test of radiation and mechanical isocenters for a medical accelerator. The following were examined: scanning repeatability and image homogeneity, the impact of image processing on data processing in coincidence tests, and irradiation conditions—monitor units per radiation beam and irradiation field are selected. Optimal conditions for carrying out the test are chosen: (i) the multi-leaf collimator gap should preferably be 5 mm for 2D star shot irradiation, (ii) it is recommended to apply ≥2500–≤5000 MU per beam to obtain a strong signal enabling easy data processing, (iii) Mean filter can be applied to the images to improve calculations. An approach to dosimeter reuse with the goal of reducing costs is presented; the number of reuses is related to the MUs per beam, which, in this study, is about 5–57 for 30,000–2500 MU per beam (four fields). The proposed reusable system was successfully applied to the coincidence tests, confirming its suitability as a new potential quality assurance tool in radiotherapy.

## 1. Introduction

Radiotherapy is a treatment possibility for patients suffering from cancer. It employs ionizing radiation and cutting-edge technology to target large and small cancer volumes with high spatial accuracy by means of stereotactic radiosurgery (SRS) and stereotactic body radiation therapy (SBRT) [1,2]. The most important aspect is increasing the dose of radiation directed to cancer cells while sparing healthy tissue. To achieve this goal, each step of radiotherapy planning and irradiation should be carried out adhering to rigorous procedures of quality assurance (QA) [3,4]. This work focuses on one QA procedure related to the coincidence test of radiation and mechanical isocenter of a medical accelerator [5] and the development of a 2D method for such an application. The method consists of a radiochromic gel dosimeter in a cuboidal container, scanning with a flatbed scanner, and a dedicated software package for processing data.

Current QA procedures for the coincidence test rely mostly on a few commercial methods employing the Winston–Lutz test [6] or star shot measurements [7,8,9] using 2D flat dosimeters and electronic portal imaging devices (EPIDs) [10,11,12,13,14,15]. However, several new approaches to performing such tests have recently been explored and may increase the number of potentially useful techniques. These include the use of 3D polymer gel dosimeters (dosimeter acronyms: NIPAM, MAGIC, PABIG^nx^, PAGAT, MAGAT, VIP), and one radiochromic gel dosimeter (PVA-I) (Table 1). Most of these dosimeters are irradiated to produce a 2D star pattern. In turn, polymer gel dosimeters allow the expansion of star shot irradiation into 3D space. After irradiation, dosimeters are measured by 2D or 3D imaging techniques (Table 1). The data are processed using various software packages; for instance, for 2D calculations, myQA Machines (IBA, Schwarzenbruck, Germany), DoseLab (Varian, Palo Alto, CA, USA), FilmQA Pro™ (Ashland, Westmont, IL, USA), Mephisto (PTW, Freiburg, Germany), and polyGeVero-CT (v. 1.2, GeVero Co., Lodz, Poland), and for 3D calculations, polyGeVero-CT (v. 1.2, GeVero Co., Lodz, Poland) (Table 1).

**Figure 1:**
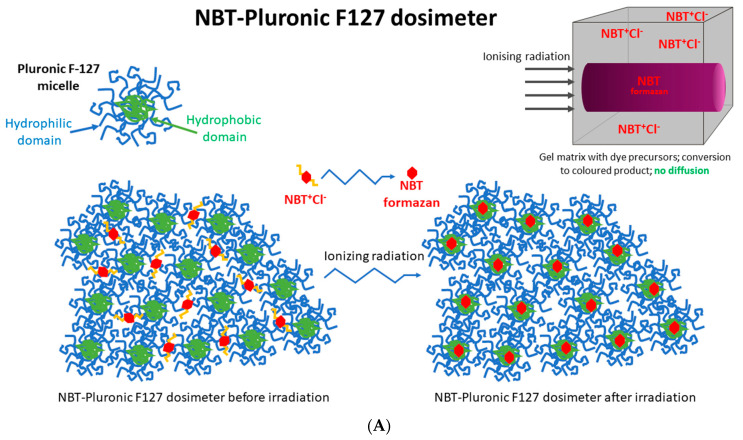

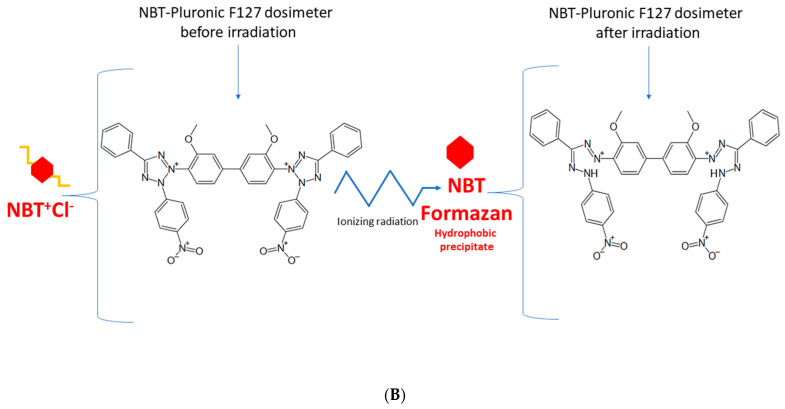
Schematic illustrating the chemical structure (**A**) and reactions (**B**) in the NBT–Pluronic F-127 dosimeter. In (**A**), the dosimeter is shown as a physical gel made of Pluronic F-127 copolymer forming micelles comprising hydrophobic and hydrophilic domains. Hydrophilic nitro-blue tetrazolium chloride (NBT^+^Cl^-^) molecules are dissolved in water in the vicinity of the hydrophilic domains of Pluronic F-127. After irradiation, NBT^+^Cl^-^ is converted into water insoluble formazan molecules having affinity for the hydrophobic domains of Pluronic F-127; they do not diffuse in Pluronic F-127. In (**B**), the chemical reaction of NBT–Pluronic F-127 under the influence of ionizing radiation is illustrated.

All 2D and 3D dosimeters, such as film and gel dosimeters, rely on the radiation-induced responses of components sensitive to ionizing radiation. For instance, thin, flat 2D dosimeters (pos. 4, 5 in Table 1) develop a star-like color pattern after irradiation, and their optical density can be later measured with, e.g., flatbed scanners. In turn, polymer gel dosimeters develop a white color as a result of radical polymerization and crosslinking of monomeric vinyl components and can be measured in 3D using magnetic resonance imaging or computed tomography (pos. 3, 6–9 in Table 1). In this work, a 2D dosimeter is proposed as a detector in the coincidence test, which is a composition of a 3D radiochromic dosimeter based on a tetrazolium salt [26] (Figure 1). The use of tetrazolium salts in the production of 3D radiochromic gel dosimeters dates to 2017. At that time, novel dosimeters were proposed that were made of a new copolymer matrix of micelles forming poly(ethylene oxide)-*block*-poly(propylene oxide)-*block*-poly(ethylene oxide) (Pluronic F-127) [27,28,29,30,31,32,33,34] infused with 2,3,5-triphenyltetrazolium chloride (TTC). Under the influence of ionizing radiation, TTC converts into an intensely colored formazan (in the case of TTC, the color is orange-red), which is insoluble in water. The insolubility of the formazan in water means that it does not diffuse in the Pluronic F-127 matrix (Figure 1). This is a very important feature that distinguishes radiochromic dosimeters based on tetrazolium salts from other similar dosimeters, which are often made of radiation-sensitive substrates that transform into a soluble, colored product that diffuses in the matrix during storage. The solubility of the colored product in the dosimeter matrix means that information about radiation dose distribution is not retained in the dosimeter volume during storage. On the contrary, the insolubility of the colored product in the dosimeter matrix is related to maintaining the integrity of the 2D/3D dose distribution information after irradiation. From this point of view, tetrazolium salts containing radiochromic dosimeters outperform other dosimeters. In consequence, further research presented the possibility of using various tetrazolium salts as possible components of 3D radiochromic dosimeters [26]. Particular attention was paid to, among others, nitro blue tetrazolium chloride (NBT) in the Pluronic F-127 matrix (Figure 1). The main features of the NBT–Pluronic F-127 dosimeter are as follows: (i) composition of 0.0818% NBT (1 mM), 25% Pluronic F-127, and 0.136 × 10^−2^% sodium formate; (ii) insensitivity to changes in dose rate for photons; (iii) the mean dose sensitivity is 0.0047 ± 0.1 × 10^−4^ (Gy cm)^−1^; a diversion in the dose-response was seen for electrons as a source of irradiation, (iv) the linear dose range and a dynamic dose range are <1–≥150 Gy; the dose threshold is <1 Gy [26]. However, this dosimeter has never been used to test medical accelerators in routine QA procedures.

The aim of this work is to propose a reusable 2D tool for the coincidence test of radiation and mechanical isocenter of medical accelerators. The tool consists of the NBT–Pluronic F-127 radiochromic dosimeter in a cuboidal container, which, after 2D star shot irradiation, is measured in 2D using an easily accessible flatbed scanner, and the resulting data are processed using the dedicated functionality of the polyGeVero-CT software package (v. 1.2, GeVero Co., Lodz, Poland; https://www.polygevero.com/product/polyGeVero-CT-software, accessed on 26 May 2024). The study presents technical considerations regarding the preparation and operation of the dosimeter, 2D scanning (image uniformity and scanning reproducibility), and the approach to data processing. The tool is analyzed in terms of irradiation conditions, including the size of the accelerator’s multileaf collimator gaps and the number of monitor units required for proper irradiation of the dosimeter. Although the changes occurring in the dosimeter after irradiation are irreversible, this work presents an approach to reusing the dosimeter in the coincidence test. The features of the NBT–Pluronic F-127 dosimeter proposed in this work are discussed in comparison to the Fricke-XO–Pluronic F-127 dosimeter, which has recently been reported elsewhere [20].

## 2. Results

### 2.1. Scanning Reproducibly and Image Uniformity

**Figure 2:**
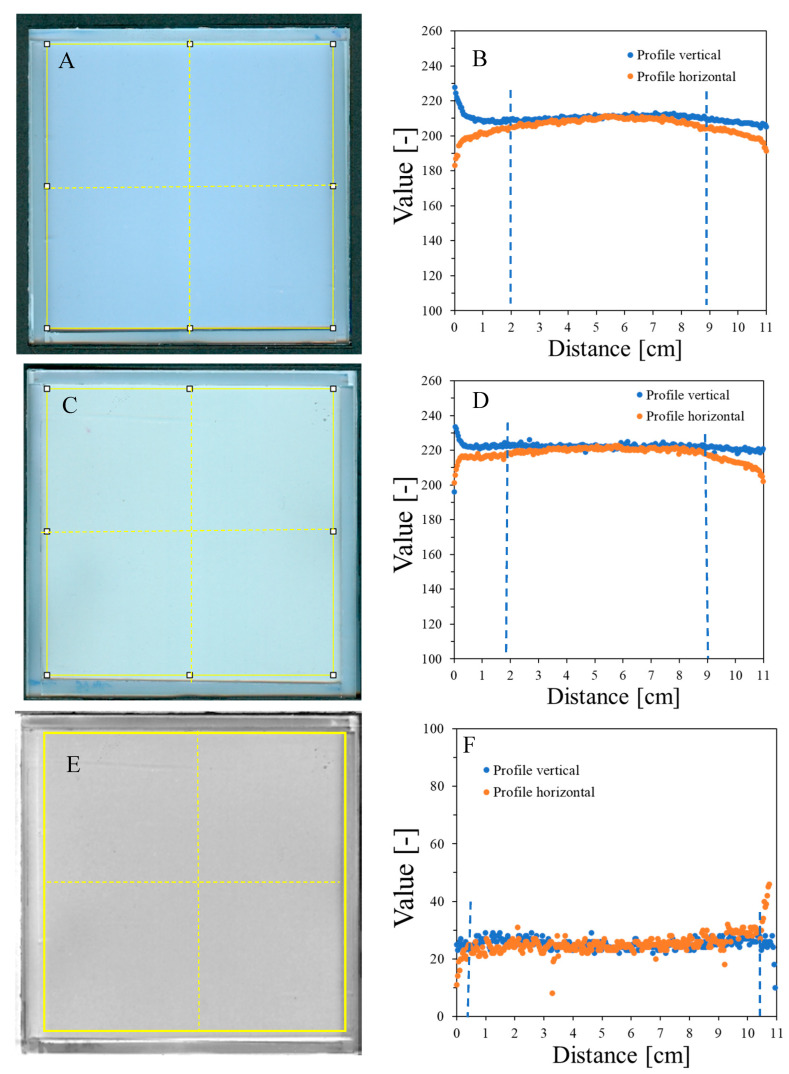
Scanning uniformity of a plane poly(methyl methacrylate) container covered with polyester foil and three sheets of 120 g/m^2^ white paper on top: image after scanning with an HP Scanjet G3010 scanner (75 dpi, scanning mode: color) (**A**) and horizontal and vertical profiles for the sample (**B**). (**C**,**D**) correspond to (**A**,**B**), respectively, with the container filled with the NBT–Pluronic F-127 dosimeter. The yellow square and dashed lines in (**A**,**C**) denote the regions used to calculate the mean values and the positions of the profiles presented in (**B**,**D**), respectively. (**E**) is the image obtained after subtracting (**A**) from (**C**) (performed in polyGeVero-CT), and (**F**) are the profiles as indicated in (**E**) with yellow dashed lines. The yellow frame in (**E**) shows the area over which the mean pixel value [-] was calculated. Blue dashed lines indicate regions of deviations from horizontal profiles.

HP Scanjet scanning reproducibility was assessed by scanning an empty container covered with transparent polyester film and three sheets of 120 g/m^2^ white paper ten times (Figure 2). Afterward, the mean pixel value [-] of the selected area (~11 × 11 cm^2^) was calculated for each sample, and for these ten samples, the mean value was also calculated, which equals 204.4 ± 4.8 [-] (Figure 2A). The percentage difference between these mean values and the mean values for each sample is in the range of 0.01–0.29%. This indicates that scanning with the selected scanner produces repeatable results. In addition, the container covered with 120 g/m^2^ paper produced high values in scanning. This is important because it propagates on a higher measurable range with the HP scanner for NBT–Pluronic F-127 in the container; measurement with an HP scanner of a container covered with black paper reduced the values to approximately 46.8 ± 4.7 [-]. In turn, unirradiated NBT–Pluronic F-127 in a container covered with three sheets of 120 g/m^2^ white paper was measured ten times to examine the repeatability of the dosimeter scanning. The mean value of 218.5 ± 3.6 [-], calculated from the mean values for each of the ten measurements, denotes the high reproducibility of the dosimeter scanning. The percentage difference between this mean value and the mean value for each sample is in the range of 0.01–0.15%.

Image uniformity was assessed by drawing horizontal and vertical profiles on the image of the scanned sample (Figure 2B). Due to the frame structure of the container, the scanner light illuminates the edges of the container, which creates shadows near the edges. This, in turn, affects the uniformity of the profiles so that they bend up (vertical profiles) or down (horizontal profiles) to a distance of ~2 cm from the edges. The mean value for the profiles is 209.9 ± 3.5 [-] (vertical profile) and 205.1 ± 5.5 [-] (horizontal profile) (standard deviations amount to 1.6 and 2.7%, respectively, of the calculated mean values) (Figure 2B). In conclusion, the deviations of the image zones close to the rims may affect the reading of the NBT–Pluronic F-127 dosimeter after 2D star shot irradiation in the coincidence test of radiation and mechanical isocenter so that in the vertical position, the values may be increased close to the rim and in the horizontal position, decreased. However, this should not have a negative impact on data processing and result calculation in the polyGeVero-CT software package—see the sections below for further discussion. Background subtraction can be considered when processing data. This is illustrated in Figure 2E,F. Shown are the results after subtracting the image presented in Figure 2A from an image presented in Figure 2C. This operation resulted in more uniform profiles close to the rims (Figure 2F) (the mean value for the area indicated in Figure 2E is 26.2 ± 3.8 [-]). In this case, ~10.5 × 10.5 cm^2^ area of NBT–Pluronic F-127 is available for irradiation (zones of ~0.5 cm from the edges can be avoided).

### 2.2. In-Time Stability of NBT–Pluronic F127

**Figure 3:**
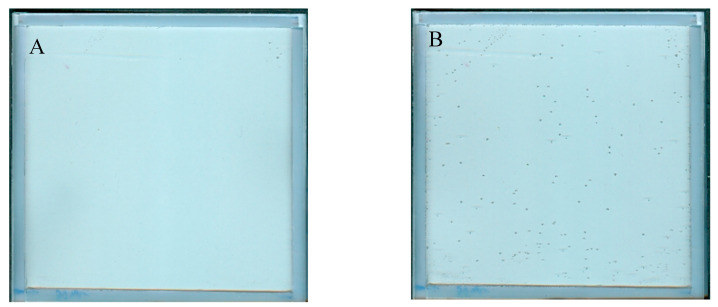

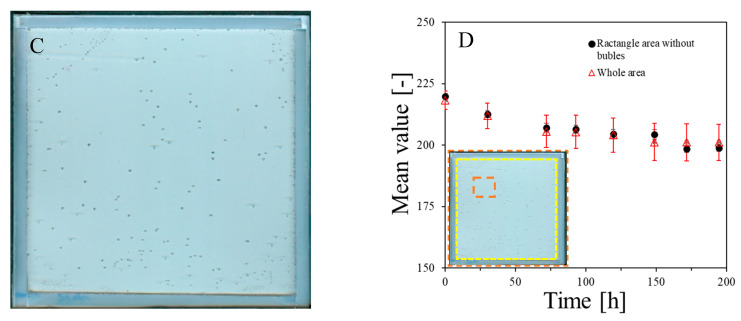
Stability of NBT–Pluronic in a cuboidal container: (**A**)—just after preparation, (**B**)—24 h, and (**C**)—48 h after preparation. In (**D**), the changes in the mean value for the regions indicated in the inset are presented versus storage time (the whole area indicated by the dashed line and the narrowed area indicated by the red dashed line).

The NBT–Pluronic F-127 dosimeter in a container with a stiff but flexible top cover made of a thin polymer foil was measured over time after preparation. The results are presented in Figure 3A–D. In Figure 3A, the dosimeter sample can be seen just after preparation as a relatively uniform image without any disturbing distortion to the gel dosimeter. However, after prolonged storage, small bubbles may appear. They are first visible about 5 h after preparation, and their number is much greater 24 h after preparation (Figure 3B). Their number appears to be constant with longer storage times (Figure 3C). The most likely cause of the appearance of bubbles is the design of a cuboidal container with a flexible, not very stably attached foil on top of the dosimeter, which is convenient for use in this work. In Figure 3D, the change in the mean value calculated for the two areas, as indicated in the inset (the whole area and a smaller area without bubbles), is plotted as a function of storage time. Storage for the first ~70 h is associated with color drift, which appears to slow down after this time. It is worth adding that this color drift is barely visible to the naked eye. This color drift may have a rather small impact on the coincidence test discussed in this work.

### 2.3. Irradiation Parameters: MU and Beam Size

**Figure 4:**
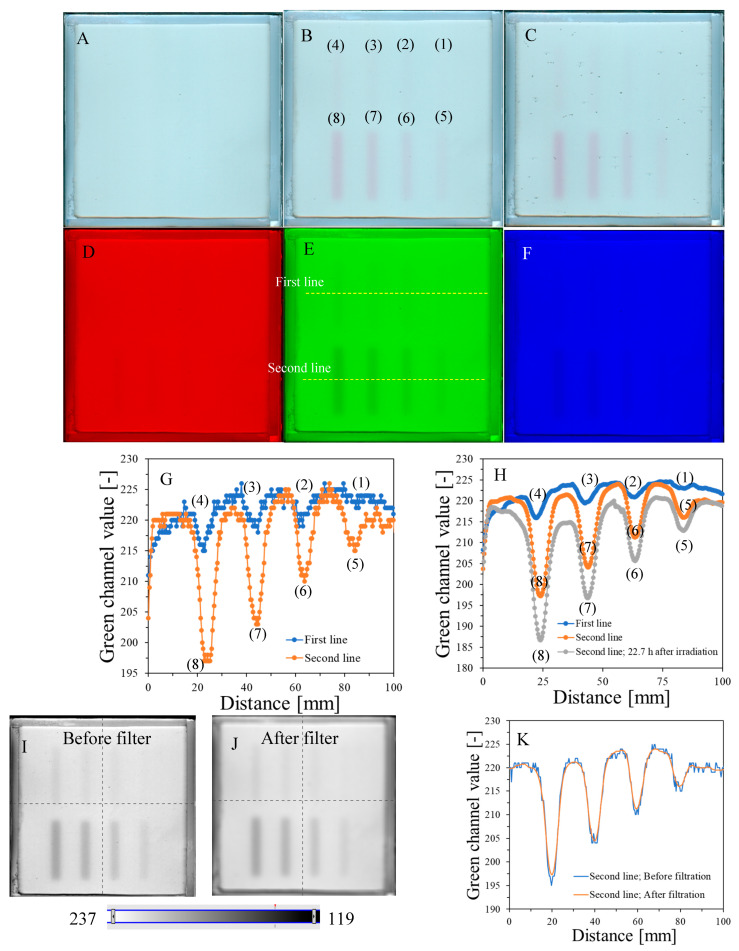

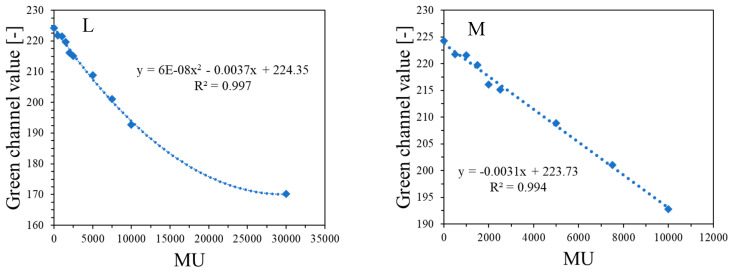
Dose-response of the NBT–Pluronic F-127 dosimeter to irradiation with the TrueBeam medical accelerator for monitor units: 500 (1), 1000 (2), 1500 (3), 2000 (4), 2500 (5), 5000 (6), 7500 (7), and 10,000 (8) MU. Irradiation pattern: 0.5 × 4 cm^2^ stripes. In (**A**), the sample just after preparation is visible. In (**B**), the same sample is visible after irradiation with eight fields of different monitor units. In (**C**), the same sample as in (**B**) can be seen, but after 22.7 h of storage. (**A**–**C**) are scans with an HP Scanjet G3010 flatbed scanner (75 dpi). (**D**–**F**) are the red, green, and blue channels of the RGB color model (for the green channel, the changes after irradiation are the most visible). (**G**,**F**) are profiles for the green channel across the irradiated zones, as indicated by the yellow dashed lines in (**E**). In (**G**), the unfiltered profiles are shown, and in (**H**), the same profiles are presented after filtering with the Mean filter (Kernel mode: 2D, Kernel size: 3, Kernel unit: mm, 1 iteration). (**I**,**J**) present two images for the green channel before and after applying the Mean filter, and (**K**) is for two superimposed profiles for the image in (**E**) before and after the Mean filter was applied. In (**L**), the relation of the green channel value versus monitor units is shown. Note that the point value at 30,000 MU in (**L**) was taken after analysis of the data related to NBT–Pluronic F-127 star shot irradiation from the region of overlapping four 7500 MU beams (see Figure 6). (**M**) refers to the same relationship as in (**L**), but for a narrower range of MUs and using linear regression.

**Figure 5:**
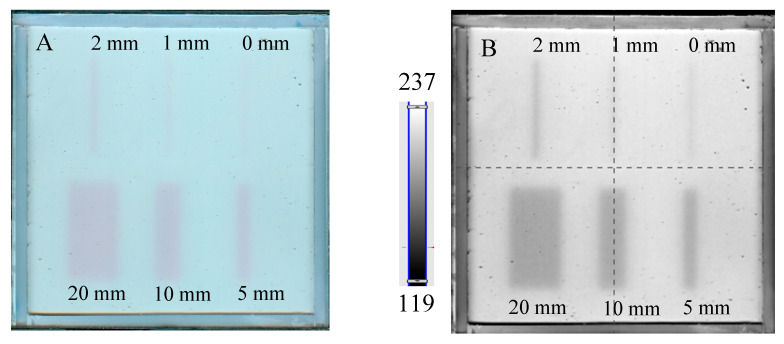

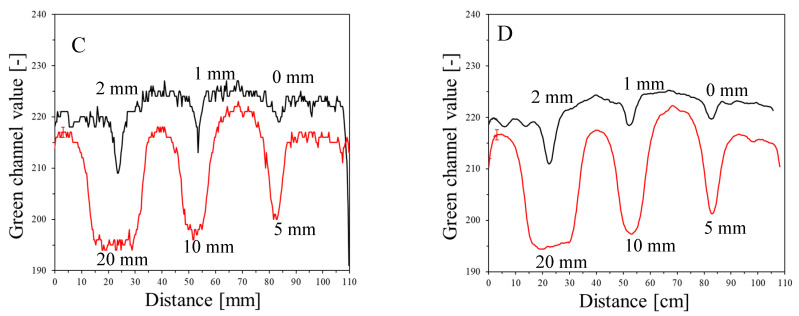
Dose-response of the NBT–Pluronic F-127 dosimeter to irradiation with the TrueBeam medical accelerator for 7500 MU per beam and various beam sizes (irradiation fields): 0, 1, 2, 5, 10, and 20 mm. (**A**)—image after scanning with an HP Scanjet scanner, (**B**)—image of the green channel of the RGB color space, (**C**)—profiles across the irradiated regions in (**B**) (no filter applied) and (**D**)—profiles across the irradiated regions in (**B**) after a Mean filter applied (Kernel mode: 2D, Kernel size: 3, Kernel unit: mm, 1 iteration). Black and red profiles (in **C**,**D**) correspond to upper and lower irradiated regions, respectively, in (**B**).

The response of the dosimeter was tested by irradiating with the TrueBeam accelerator at two different settings, one by irradiating it with different monitor units and the same size of irradiated regions (Figure 4) and the other by irradiating it with one arbitrarily chosen monitor unit (7500 MU) and different sizes of irradiated regions, which was realized by changing the MLC gap (Figure 5). The results of the unirradiated and irradiated dosimeter just after exposure and 2D scanning with the HP Scanjet G3010 are presented in Figure 4A,B and Figure 5A,B. Parts of the dosimeter that have been irradiated turn pink-red due to the conversion of nitro-blue tetrazolium chloride to water-insoluble formazan. Due to the insolubility of formazan in water, the developed color is permanent and does not diffuse in the gel dosimeter matrix over time of storage outside the irradiated region. Therefore, there is no need to 2D scan the dosimeter immediately after exposure; the dosimeter can be scanned many hours or even days after irradiation. However, the construction of the container developed in this work may facilitate the formation of tiny bubbles over time of storage (Figure 4C), which may slightly affect the calculations in polyGeVero-CT. In the future, this could be remedied by improving the design of the container. The dosimeter also ages (Figure 4C,H), as written in the previous section; however, this is not a significant problem that would affect the processing of coincidence test data. The green channel of the RGB color model has the largest contribution to the observed color of irradiated regions (Figure 4D–F). Consequently, it was further used in the processing of the coincidence test data.

Figure 4G,H are profiles across all irradiated regions before (Figure 4G) and after (Figure 4H) applied filtration (Mean filter), whereas Figure 4I,J show the images of samples before and after filtration and, in Figure 4K, two profiles for the chosen irradiated regions are superimposed to facilitate the analysis of the impact of this operation on the shape of profiles. The conclusions are that filtering smoothed the images and slightly reduced image noise. For instance, the mean value for the whole dosimeter image is 217.9 ± 8.5 and 217.9 ± 7.9 [-] for the unfiltered and filtered image (Mean filter, Kernel 7 mm, 1 iteration), respectively. However, the shape of profiles remains the same as before filtration for Kernel 1–3 mm. It was also learned that the filtration for the Kernel size of 3 mm is optimal, and for higher Kernel size (5–7 mm), distortions of the profiles were observed. In Figure 4L,M, the response of the system to irradiation is presented in the MU range; green channel values are calculated as the mean of the irradiated regions, and the low standard deviation values are covered by the points. The relationship is linear up to 10,000 MU, and at about 30,000 MU, the dosimeter becomes saturated in color. In summary, image filtering (Mean filter, Kernel 3 mm) was considered as an option to facilitate the calculations related to the coincidence test. Most importantly, however, the results point to 1500–10,000 MUs per beam as a likely range that could be applied to the dosimeter during star shot irradiation—the coincidence test.

The results of the irradiation according to the second pattern (different beam sizes (fields of irradiation) from different MLC gaps at one MU = 7500) are presented in Figure 5. In Figure 5A, an image about 30 min after irradiation and HP Scanjet scanning is presented, and the corresponding green channel of RGB is shown in Figure 5B (represented in greyscale). Profiles before and after filtration (Mean filter) are shown in Figure 5C,D. It can be seen that the dosimeter registers irradiation even for an MLC gap = 0 mm. Theoretically, any MLC gap can be used to carry out the coincidence test, particularly after image filtering. Without image filtration, the gap should be above 1 mm. However, taking into account the size of the dosimeter and the results in Figure 4 and Figure 5 (changes in the dosimeter after exposure), a 5 mm gap was adopted for the coincidence test.

### 2.4. Coincidence of the Radiation and Mechanical Isocenters

**Figure 6:**
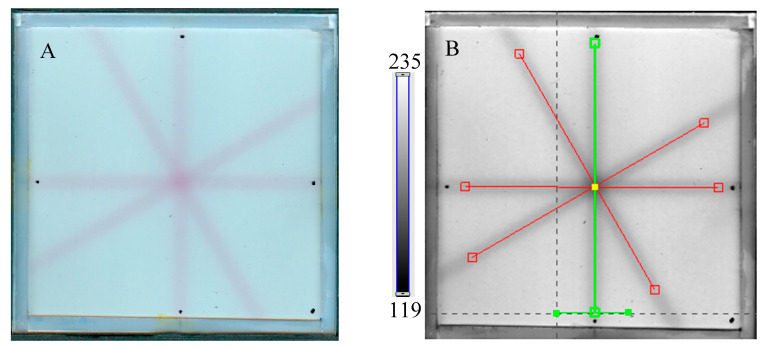

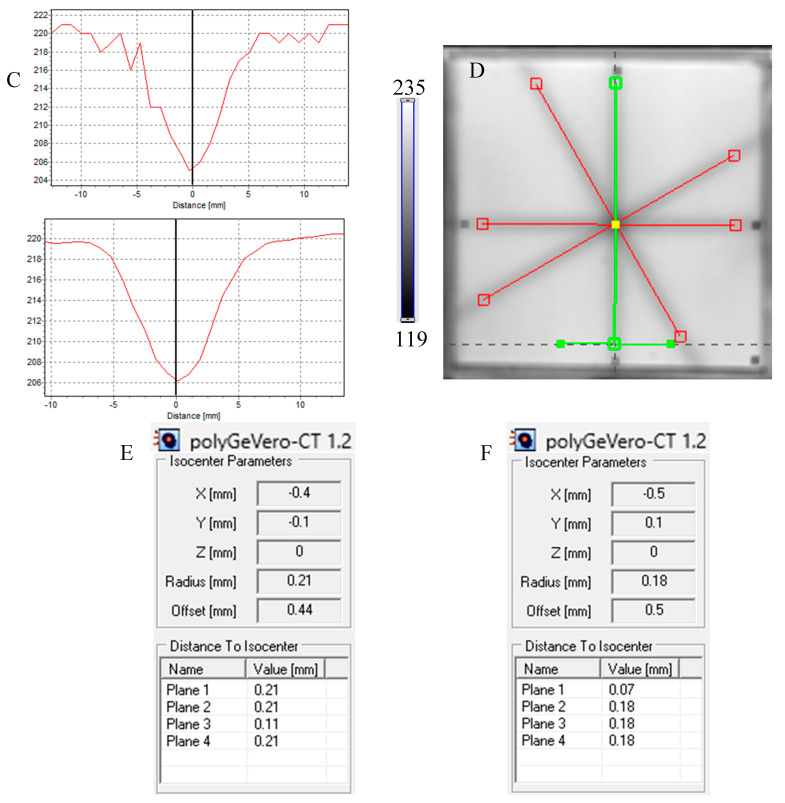
Response of the NBT–Pluronic F-127 dosimeter to irradiation with the TrueBeam medical accelerator according to the star shot pattern: the coincidence test of the mechanical and irradiation isocenters. The number of monitor units per beam was 7500 MU. (**A**)—image after scanning with an HP Scanjet scanner, (**B**)—image of the green channel of the RGB color model viewed in the polyGeVero-CT with tools (red and green lines) specific for the coincidence test, (**C**)—profiles at one end of the irradiation area—green line perpendicular to the irradiated region in (**B**): upper profile is for the image before applying the Mean filter and the lower profile is for the same profile but for the image after applying the Mean filter (Kernel mode: 2D, Kernel size: 3, Kernel unit: mm, 1 iteration), (**D**)—corresponds to the image in (**B**), however, after applying the Mean filter, (**E**,**F**)—calculation results for the coincidence test: (**E**) refers to the image in (**B**,**F**) is for the image in (**D**). The black dots on the irradiated regions in (**A**,**B**,**D**) are markers used for the indication of a mechanical isocenter in polyGeVero-CT (Offset).

The study related to the application of the dosimeter to the coincidence test of radiation and mechanical isocenters of the TrueBeam accelerator was performed in the following order. First, the test was conducted for the selected irradiation settings: 2D star shot irradiation, 7500 MU per beam, 4 beams, and a 5 mm MLC gap. The results, 2D HP Scanjet images, were processed for unfiltered and filtered images (Figure 6A–F). 

**Figure 7:**
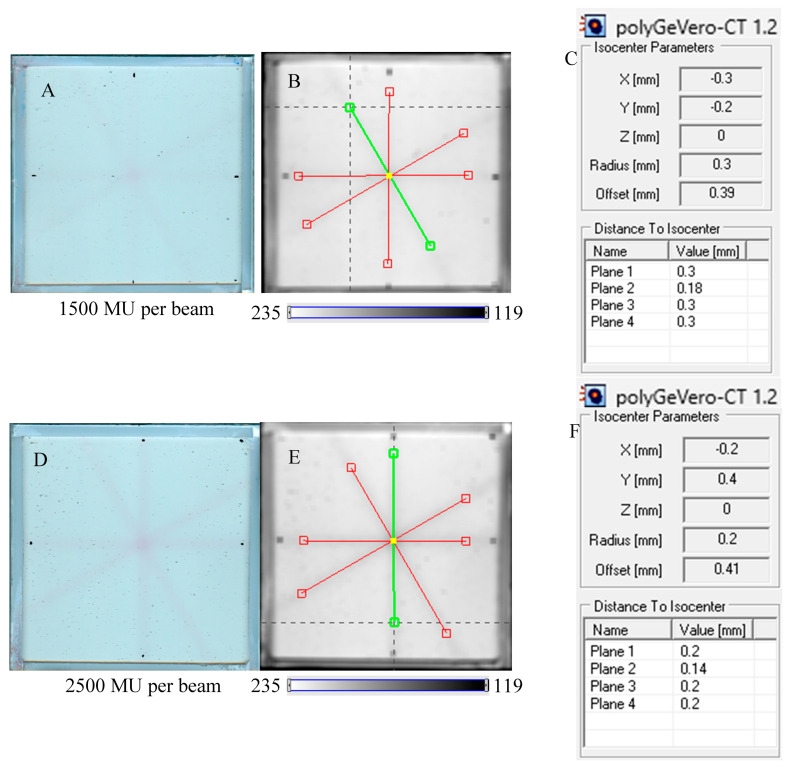

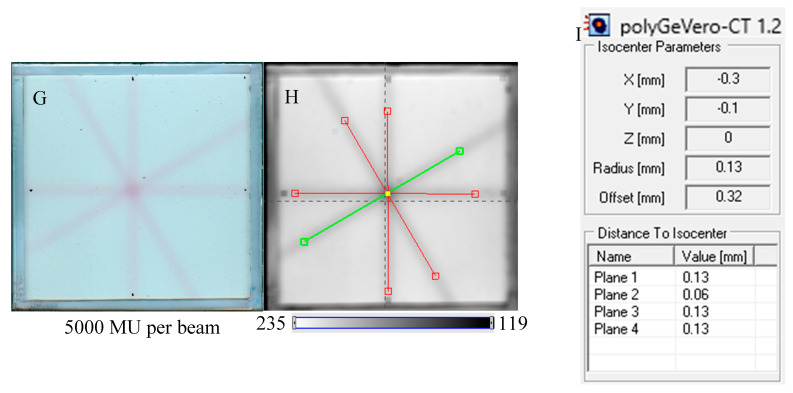
Response of the NBT–Pluronic F-127 dosimeter to irradiation with the TrueBeam medical accelerator according to the star shot pattern: the coincidence test of the mechanical and irradiation isocenters. The number of monitor units per beam was 1500 (**A**–**C**), 2500 (**D**–**F**), and 5000 MU (**G**–**I**). (**A**,**D**,**G**) are images after scanning with an HP Scanjet scanner, (**B**,**E**,**H**) are images of the green channel of RGB color space viewed in the polyGeVero-CT with tools (red and green lines) specific for the coincidence test (images filtered with the Mean filter: Kernel mode: 2D, Kernel size: 3, Kernel unit: mm, 1 iteration). (**C**,**F**,**I**) are the calculation results of the coincidence test. The black dots on (**A**,**D**,**G**) are markers used to indicate the mechanical isocenter in polyGeVero-CT (Offset).

Next, the test was performed for different MU values per beam to determine the lowest possible (Figure 7) with respect to the findings presented in the former section. In Figure 6A,B,D, the images after star shot irradiation and scanning with an HP Scanjet scanner can be viewed. In all images, a specific star shot red-pink pattern is visible, corresponding to color formation after irradiation. Figure 6A is the image about 30 min after irradiation, whereas Figure 6B is the green channel of the RGB color space with red and green lines of the tools in the polyGeVero-CT software for the coincidence test, and Figure 6D shows the same image as in B, but after applying filtering. When polyGeVero-CT for the coincidence test is used, the beam profiles are drawn, as seen in Figure 6C. For unfiltered images, the profiles are slightly noisy, and the determination of the middle of the profile can be somewhat burdened. For filtered profiles, this problem is much avoided, and data processing is facilitated. For the case considered in Figure 6, the calculation results (Figure 6E,F) are largely similar and are within the tolerance limits for the star shot measurement: ±1 mm [4].

In Figure 7, the results of the star shot irradiation of the NBT–Pluronic F-127 dosimeter in cuboidal containers are presented for 1500 (Figure 7A–C), 2500 (Figure 7D–F), and 5000 MU (Figure 7G–I) per beam. For each irradiation condition, both the photographs after irradiation, images of the green channel (RGB color space), and the results of the coincidence test calculations are presented. The following conclusions stem from this experiment. It was possible to process data for the green channel images in the polyGeVero-CT software package for each irradiation condition. The simplest data processing was for 5000 MU per beam due to the strong signal. In turn, the irradiation with the 1500 MU per beam is considered as the lower threshold for this tool: NBT–Pluronic F-127 in the cuboidal container and 2D scanning with an HP Scanjet scanner. All results are within the tolerance limits: ±1 mm [4] for irradiation set at 7500 MU per beam, as stated above.

The coincidence of planned and measured MLC angles was calculated for the irradiation settings examined in this work. The results are presented in Table 2. In the cases examined for the star shot irradiation settings of 1500–7500 MU per beam, the differences were less than ±0.5°, which denotes the planned and measured MLC angle values are similar within a tolerance of ±0.5° [35]. The study proved that the new 2D dosimetric system is useful for the coincidence tests and demonstrated the correct operation of the accelerator through the close overlap of mechanical and radiation isocenters and planned and measured MLC angles.

### 2.5. Reusability of the Dosimeter

The approach to reusing the NBT–Pluronic F-127 dosimeter is based on the chemical nature of this dosimeter, as illustrated in Figure 1. Before irradiation, the water-soluble NBT molecules are well dissolved in the aqueous solution of the Pluronic F-127 copolymer. This copolymer forms micelles with a hydrophobic inner core (propylene oxide units) and external hydrophilic domains (ethylene oxide units) (Figure 1). A feature of such micelles is the possibility of solubilization of hydrophobic chemicals inside the hydrophobic inner cores. Upon irradiation, NBT converts to NBT formazan, which is insoluble in water. In the absence of micelles, NBT formazan precipitates, as seen elsewhere (consult Figure 4 in [36]), which would be an undesirable effect for a reusable dosimeter. However, this does not apply to the Pluronic F-127-based dosimeter. It was assumed and later verified that in the presence of Pluronic F-127 micelles, the hydrophobic NBT formazan molecules are homogeneously solubilized in the NBT–Pluronic F-127 dosimeter. Organoleptic observations did not indicate any precipitate formation in the dosimeter after irradiation, melting, and mixing the liquid dosimeter. The NBT formazan molecules were evenly distributed in the dosimeter substance without any aggregates being visible. These observations were used to propose an approach to reuse the NBT–Pluronic F-127 dosimeter.

**Figure 8:**
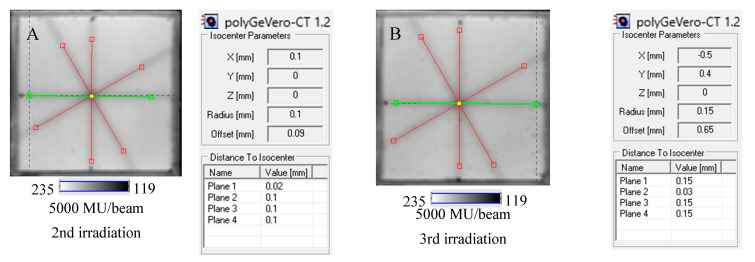

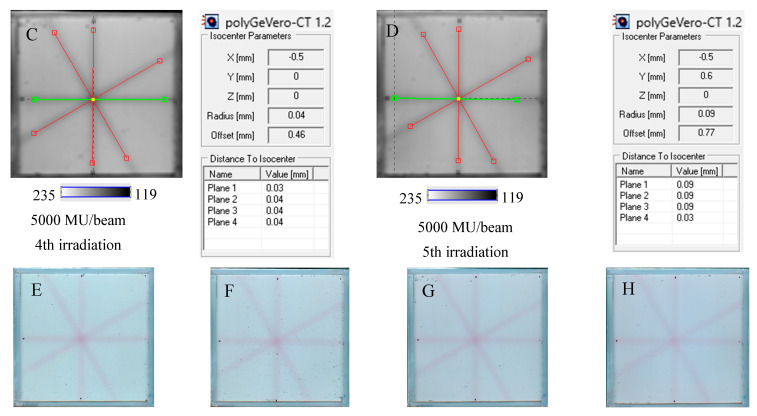
Reusability of the tool for the coincidence test: NBT–Pluronic F-127 dosimeter in a cuboidal container and 2D scanning with an HP Scanjet scanner. One container with the dosimeter was used. After each 2D star shot irradiation of the NBT–Pluronic F-127 dosimeter (5000 MU/beam) and HP Scanjet scanning, the dosimeter was melted at about 4 °C and reused. (**A**)—used for the second time, (**B**)—used for the third time, (**C**)—used for the fourth time, (**D**)—used for the fifth time. (**E**–**H**) are HP Scanjet images after the 2nd, 3rd, 4th, and 5th use for the coincidence test, respectively.

The NBT–Pluronic F-127 dosimeter in a cuboidal container has been reused several times to illustrate this application approach. After each irradiation, the dosimeter was melted at about 4 °C for 5 min. Before subsequent irradiation, it was kept at room temperature for 5 min, which was sufficient to convert the solution into a physical gel. The dosimeter was irradiated periodically with an interval amounting to one or more days between irradiations, as indicated in Section 4.5. The results are presented in Figure 8. In all cases presented in this figure, the calculations performed in polyGeVero-CT were quick and easy due to the clear image of the star shot pattern visible despite the dosimeter being irradiated several times. Because each irradiation produces a pink-red star shot pattern (colored NBT formazan molecules), the melting and mixing of the molten dosimeter propagate onto a higher color intensity of the bulk dosimeter before the subsequent irradiation. This increase in color was almost invisible to the naked eye; however, it was calculated that the color change amounted to about 5% for samples irradiated between two and five times. Like the above-presented results, the parameters of the test are within the tolerance limits: ±1 mm [4].

The reusability of this dosimeter can be roughly estimated based on the following information: (i) a saturation of the dosimeter at about 30,000 MU (Figure 4L), (ii) a volume of the dosimeter of about 38 cm^3^, (iii) a volume of all four irradiation areas equal to about 7.9 cm^3^, and (iv) percentage consumption of the dosimeter for a particular MU value. To summarize, reusability can be expressed by the relation of No. of uses = 142,962 × MU^−1^. For instance, if the dosimeter is irradiated with 4 fields of ~0.5 cm width and 30,000 MU per beam, it can only be used ~5 times. However, if it is irradiated in the same way but for a MU number that is 2500, then it can be used ~57 times in the coincidence test. Note that this estimate indicates the NBT molecules available in the system for irradiation—conversion to NBT formazan. However, it should also be emphasized that the adopted method of repeated use of the dosimeter will generate an increased background from dissolved pink-red NBT formazan molecules in Pluronic F-127 micelles after irradiation and melting at low temperatures. In such cases, an additional data processing procedure of background subtraction before calculating the parameters of the test in the polyGeVero-CT software package may be applied. Background subtraction was not obligatory in this study because of a fewer number of repetitions. A larger number of repetitions of the coincidence test were not performed due to limited access to the accelerator.

### 2.6. Measurement Uncertainty

The use of a tool for examining the coincidence of the radiation and mechanical isocenter, NBT–Pluronic F-127 in a cuboidal container in combination with a flatbed scanner and data processing using the polyGeVero-CT software package, is associated with the following components of total uncertainty: (i) positioning of the NBT–Pluronic F-127 dosimeter container on the accelerator bench at its mechanical isocenter, which amounts to <1 mm, using the LAP laser system, (ii) precision related to data (images) processing in the polyGeVero-CT software—setting the origin (mechanical isocenter) using markers made with a pen on images of the scanned container, which is 0.34 mm; the accuracy of defining lines along beams in images (see, e.g., Figure 8—red and green lines), which is 10^−8^ mm; for four lines and software algorithm it is 8 × 10^−8^ mm; and the precision of the calculations performed by the software, which is 10^−8^. In summary, the combined standard uncertainty [37,38] is 1.06 mm. It should be noticed that the coincidence test using the tool proposed in this work allows detecting the radiation isocenter as a circle with a radius > 0 mm and even as a point with radius = 0 mm [21]. They are much smaller than the recommended tolerance of ±1 mm [4]. The combined uncertainty can be reduced by optimizing the container for the NBT–Pluronic F-127 dosimeter by using thinner mechanical isocenter markers on the container and by using a positioning system with lower uncertainty than the LAP system.

## 3. Discussion: NBT–Pluronic F-127 vs. Fricke–XO–Pluronic F-127

To date, we have investigated two radiochromic gel dosimeters as 2D systems for routine medical linear accelerator (X-ray radiation) testing–coincidence of mechanical and radiation isocenters: NBT–Pluronic F-127 in current work and Fricke–XO–Pluronic F-127 reported elsewhere [20]. There is only one similarity between these dosimeters: the Pluronic F-127 matrix. In both cases, it allows easy production of dosimeters by dissolving other components at low temperatures (e.g., ~4 °C) in the Pluronic F-127 solution, which quickly converts into a physical gel at a temperature of approximately 17 °C in less than 5 min. The main reactions occurring under the influence of ionizing radiation are different for these two dosimeters and consist of the formation of colored products from the NBT and Fe^+2^ + XO for NBT–Pluronic F-127 and Fricke–XO–Pluronic F-127, respectively. The chemical properties of the resulting products (NBT formazan and [Fe-XO]^+3^) translate into the stability of dosimeters and irradiated zones, as well as application features such as the number of monitor units of irradiation, maximum period of 2D scanning, and the possibility of reusing dosimeters. The most attractive feature of NBT–Pluronic F-127 is the stability of the irradiated zones, which do not diffuse after irradiation. This, in turn, translates into a 2D scanning period that can be at least 22.7 h after irradiation (the maximum period studied, however, appears to be much longer) and reusability, which translates into lower chemical consumption, cost reduction, and saving the environment. Unlike the NBT–Pluronic F-127 dosimeter, the Fricke–XO–Pluronic F-127 is significantly less stable after preparation and irradiation. After preparation, the entire gel dosimeter changes color from light yellowish-brownish to dark blue-purple, especially when the sides of the dosimeter in the plastic frame are in contact with air (see Figure 2 in [20]). This conversion can be observed with the naked eye approximately 2.5 h after preparation and storage at room temperature. Also, the irradiated dark blue zones are unstable and disperse over time after irradiation. This can be seen with the naked eye just 2.5 h after irradiation and storage at room temperature (see Figure 2 in [20]). Therefore, the Fricke–XO–Pluronic F-127 cannot be used as a reusable dosimeter. However, this does not disqualify it from use in radiotherapy dosimetry on account that 2D scanning is performed shortly after irradiation and preparation [20]. The advantage of Fricke–XO–Pluronic F-127 is a strong signal after irradiation even with 500 MU (see Figure 2 in [20]). This is in contrast to the NBT–Pluronic F-127 reaction, for which the irradiation effect is hardly visible at this MU number. Therefore, the exposure time needed to obtain the appropriate signal and then the reading quality of the NBT–Pluronic F-127 is longer than that of the Fricke–XO–Pluronic F-127 dosimeter. Consequently, further research toward the optimization of Fricke–XO–Pluronic F-127 is justified.

## 4. Materials and Methods

### 4.1. Preparation of 2D Dosimeter

**Figure 9:**
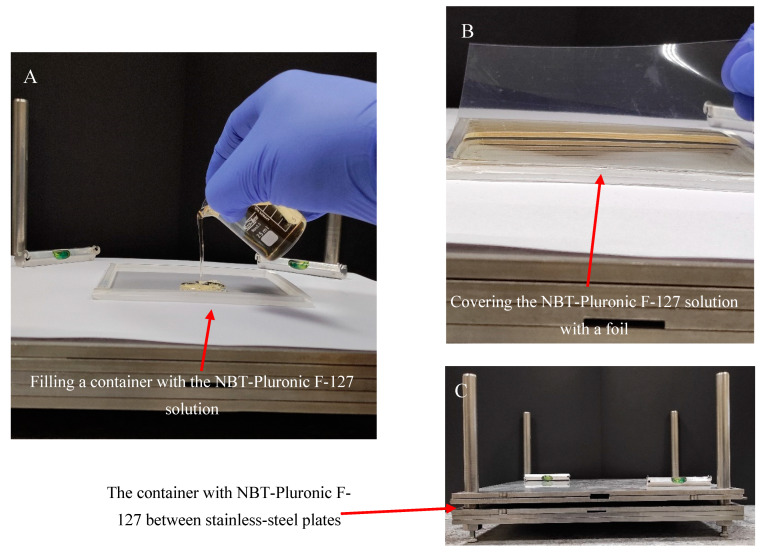
Scheme of manufacturing the NBT–Pluronic F-127 dosimeter in a cuboidal container. (**A**): Filling the container with cold (~4 °C) NBT–Pluronic F-127 dosimeter solution; the container is placed on a homemade stainless-steel device consisting of surgical stainless-steel plates. (**B**): Covering the container filled with the dosimetric solution with a stiff plastic foil. (**C**): The container with the dosimetric solution covered with foil is loaded with stainless-steel plates for ~5 min until the dosimetric solution solidifies.

Gel dosimeter samples based on 25% (*w*/*w*) Pluronic F-127 (Sigma-Aldrich, Saint Louis, MO, USA) matrix with 0.0818% (1 mM) nitro-blue tetrazolium chloride (NBT, M_w_ = 817.65 g mol^−1^) (Sigma-Aldrich) and 0.136 × 10^−2^ % sodium formate (68.01 g mol^−1^, Chempur, Piekary Śląskie, Poland) were manufactured in air as previously described [26]. After manufacturing, the gel solution was kept in a refrigerator at about 4 °C. A poly(methyl methacrylate) container of 12.5 × 12.5 × 0.6 cm^3^ was placed on a homemade device for preparing various flat materials (Figure 9). The device is made of stainless-steel plates with dimensions of 30 × 30 × 5 cm^3^ and a frame with bars. The plates can be stacked on top of each other and separated from each other using metal washers of a specific thickness. The container was placed on the stainless-steel plates, as shown in Figure 9A, and filled with the gel solution. A 0.2 mm thick transparent film (made of poly(vinyl chloride)) was then placed on top of the gel solution (Figure 9B), and the container was pressed down with two stainless-steel plates (Figure 9C). Pressing removed excess gel solution from the container and formed a uniform physical gel in the plastic container. The gel solution was converted into a physical gel in approximately 5 min. The gel dosimeter container was then ready for experiments. It was stored at room temperature (21–23 °C) without access to daylight.

### 4.2. 2D Scanning

A flatbed HP Scanjet G3010 scanner (Hewlett-Packard, Palo Alto, CA, USA) was used in this study to scan NBT–Pluronic F-127 in a cuboidal container. The reproducibility of the scanning was tested by scanning (ten times) two sheets of white A4 paper placed on the scanner bed (80 g/m^2^, MMBloom, MM Kwidzyn, Poland). The obtained mean value is equal to 254.98 ± 0.25 [-] for A4 size area, which denotes high uniformity of scanning of white paper.

The same scanner was used to scan a cuboidal container with the NBT–Pluronic F-127. The container was covered with three sheets of white paper with a grammage of 120 g/m^2^ (POL Effect, International Paper, Kwidzyn, Poland). The settings were as follows: 75 dpi, brighten/darken option: 35 (brighten), −69 (shadows), 0 (intermediate shades), sharpen the image: none, color adjustment: none (100% saturation of color), automatic correction of color: none. Scanning reproducibly and image uniformity were assessed for empty containers, as discussed in this work.

### 4.3. Stability of Non-Uniformly Irradiated Dosimeter

NBT–Pluronic F-127 was prepared in a cuboidal container, as described in Section 4.1. It was stored at room temperature (21–23 °C) and protected from daylight throughout the stability assessment period. During this time, periodical scanning was performed using an HP Scanjet G3010 scanner (75 dpi, brighten/darken option: 35 (brighten), −69 (shadows), 0 (intermediate shades), sharpen the image: none, color adjustment: none (100% saturation of color), automatic correction of color: none).

### 4.4. Ionising Radiation Irradiation

**Figure 10:**
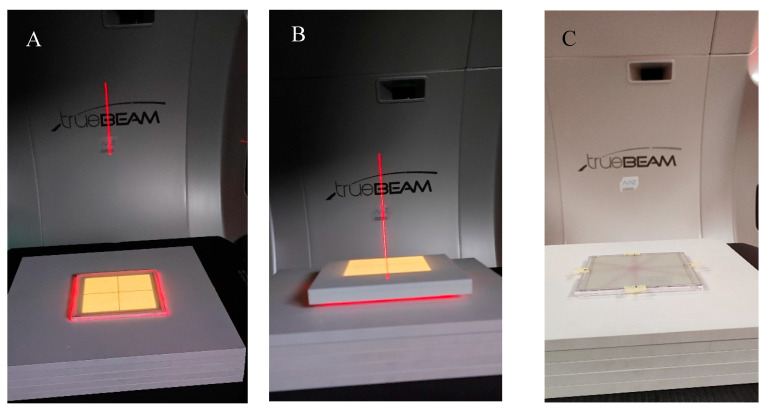
View of the irradiation set-up for the coincidence test of the mechanical and radiation isocenter of the TrueBeam accelerator. (**A**)—photograph of the NBT–Pluronic F-127 flat container placed on the TrueBeam bench and RW3 plates, (**B**)—container covered with RW3 plates, (**C**)—upper RW3 plates are removed, and the NBT–Pluronic F-127 dosimeter after irradiation is presented; a star shot irradiation pattern is visible. In A and B, the dosimetry system is visible during alignment using the LAP laser system (red laser lines) and positioning the system in a mechanical isocenter of the TrueBeam. Yellow illumination of the dosimeter comes from the light field alignment with the dosimeter area.

To investigate the possible irradiation conditions of the dosimeter to obtain appropriate images after 2D readout for data processing and the coincidence test in the polyGeVero-CT software package, the dosimeter was irradiated with different monitor units (MUs) and high-definition multi-leaf collimator (HD MLC) gaps of the TrueBeam accelerator (Varian, Palo Alto, CA, USA). For 2D reading, an HP Scanjet G3010 flatbed scanner was used (settings as in Section 4.2). Two irradiation settings were applied: (i) HD MLC was set to 5 mm (gap), the following MUs per beam were applied: 500, 1000, 1500, 2000, 2500, 5000, 7500, and 10,000 MU, (ii) HD MLC was set to obtain gaps of 0, 1, 2, 5, 10, and 20 mm and one monitor unit per beam with a value of 7500 MU was used. Additional TrueBeam settings were as follows: X-rays, 10 MV FFF, monitor unit rate of 2400 MU/min, jaws size of X: 4 cm, Y: 3 cm, gantry and collimator set to 0°. Before irradiation, a dosimeter was placed between the plates of the SP34 RW3 phantom (IBA, Schwarzenbruck, Germany); five slabs, each 1 cm thick, were placed on the accelerator bench, on which a dosimeter was placed in the accelerator isocenter and covered with two slabs of 1 and 0.5 cm thickness (Figure 10). The accelerator isocenter was set on the surface of the dosimeter, SSD 100 cm, and the surface of the dosimeter was perpendicular to the central axis of the radiation beam. 

To study the coincidence of radiation and mechanical isocenter of the TrueBeam accelerator, the dosimeter was irradiated with a 2D star shot pattern. The following settings were applied: 5 mm gap (HD MLC), gantry set to 0°, and collimator angles set at 0, 90, 150, and 240°. Various numbers of monitoring units per beam were used to investigate the minimum MU values per beam possible to irradiate and easily process the data in the polyGeVero-CT software package. The dosimeter was placed between the plates of the SP34 RW3 phantom, as detailed above. The remaining settings of the TrueBeam accelerator were as follows: X-rays, 10 MV FFF, monitor unit rate of 2400 MU/min, jaws size of X: 2 cm, Y: 20 cm, SSD 100 cm, the surface of the dosimeter was perpendicular to the central axis of the radiation beam, and the accelerator isocenter was set at dosimeter surface; the container was placed in the isocenter of the accelerator using the LAP laser system (LAP GmbH Laser Applikationen, Lüneburg, Germany).

### 4.5. Reusability Study

NBT–Pluronic F-127 was prepared in a cuboidal container, as described in Section 4.1. Before irradiation, it was stored at room temperature (21–23 °C) and protected from daylight. The approach to examining reusability was as follows. A dosimeter sample was used. It was irradiated according to the star shot pattern, 5000 MU per beam was applied (see Section 4.4), scanned with an HP Scanjet scanner (see Section 4.2), and the data were processed with the polyGeVero-CT software package (see Section 4.6). After HP Scanjet scanning, samples were stored in a refrigerator at about 4 °C until the next experiment. Refrigeration for just 5 min turned the physical gel into a viscous solution. Before subsequent irradiation, the samples were removed from the refrigerator, uncovered, and gently mixed to homogenize the structure after previous irradiation. Note that before mixing, the star shot pattern of the irradiated region was still visible but blurred. Once the solution was homogenized, the container was covered with foil and pressed with stainless steel plates, as shown in Figure 9. After about 5 min of pressing, the sample was ready for irradiation. This procedure was repeated to illustrate the possibility of reusing the dosimeter in the coincidence test. The arbitrarily chosen gaps between the successive experiments amounted to (i) 2 days between the first and second irradiations, (ii) 1 day between the second and third irradiations, (iii) 4 days between the third and fourth irradiations, and (iv) 3 days between the fourth and fifth irradiations.

### 4.6. Processing of Data

After scanning the dosimeters with an HP Scanjet G3010 flatbed scanner, the data (bmp images) were processed using the polyGeVero^®^-CT software package (v. 1.2, GeVero Co., Lodz, Poland). The polyGeVero^®^-CT software was initially designed to process 3D radiotherapy dosimetry data with CT reading. However, it has also been enriched with functionalities enabling the processing of 2D and 3D images and the coincidence test of the radiation and mechanical isocenter of medical accelerators [21,22].

## 5. Conclusions

The work describes a tool consisting of a reusable 2D radiochromic dosimeter, NBT–Pluronic F-127, in a cuboidal container, coupled with scanning with a flatbed scanner and data processing using the polyGeVero-CT software package containing dedicated functionalities of the coincidence test of radiation and mechanical isocenter. The following was established: (i) the irradiation response of NBT–Pluronic F-127 is up to about 30,000 MU; linearity of the response was observed up to about 10,000 MU, (ii) the green channel of the RGB color space has the largest contribution to the resulting color after irradiation of the dosimeter, (iii) filtering images after scanning using the Mean filter (Kernel size: 3, Kernel unit: mm, 1 iteration, calculation mode: 2D) is sufficient to facilitate calculations related to the coincidence test, (iv) any MLC gap can be used to carry out the coincidence test, particularly in the case of filtered images; without image filtration, the gap should be above 1 mm; for the container used in this work, a 5 mm gap is adequate for 2D star shot irradiation, (v) 1500–10,000 MU per beam is the range that can be applied to the dosimeter during star shot irradiation; however, it is recommended to use ≥2500–≤5000 MU per beam to obtain a strong signal enabling easy data processing; it is recommended to use a high monitor unit rate to shorten the total irradiation time, (vi) the dosimeter can be reused for the coincidence test; a procedure consisting in melting the dosimeter at about 4 °C after irradiation (it takes less than 10 min), following by mixing the liquid dosimeter to evenly distribute the colored NBT molecules in the dosimeter and solidifying at above 20 °C (it takes less than 10 min); the number of reapplications is related to the MUs per beam, and for the irradiation set up in this study is about 5–57 for 30,000–2500 MU per beam (four fields). The reusability of the NBT–Pluronic F-127 significantly reduces the costs of the coincidence test compared to the use of some single-use film dosimeters.

## Figures and Tables

**Table 1:** Example 2D and 3D systems for the coincidence test of radiation and mechanical isocenter for medical accelerators proposed by November 2023.

No.	Type of System	Irradiation Pattern Used	Scanning	Data Processing	Reference
1	Winston–Lutz test coupled with film dosimeter	Star shot 2D	Flatbed scanner	Matlab	[15]
2	Electronic Portal Imaging Devices (EPIDs)	Star shot 2D	EPID	Matlab	[10,15]
3	PAGAT polymer gel dosimeter	Star shot 2D	MR	Matlab	[16,17,18]
4	Films (2D flat dosimeters)	Star shot 2D	Epson 10000XL	Matlab	[9,19]
5	Fricke-XO-Pluronic F-127	Star shot 2D	HP Scanject G3010	polyGeVero-CT	[20]
6	PABIG^nx^ polymer gel dosimeter	Star shot 2D/3D	CBCT/iCBCT	polyGeVero-CT	[21]
7	VIP polymer gel dosimeter	Star shot 2D (Star shot 3D possible)	iCBCT	polyGeVero-CT	[22]
8	MAGAT polymer gel dosimeter	Star shot 3D	MR-linac	Matlab	[19]
9	NIPAM polymer gel dosimeter	Star shot 3D	CBCT	Matlab	[23,24]
10	Radiochromic gel dosimeter: poly(vinyl alcohol)-iodide (PVA-I) + Winston–Lutz test	Star shot 3D	Flatbed scanner	Matlab	[25]

**Table 2:** Results of measured MLC angles [°] and difference: planned − measured MLC angles [°], for irradiation settings with MLC angles changed during the test of coincidence of radiation and mechanical isocenters using NBT–Pluronic F-127 in a cuboidal container, an HP Scanjet 2D scanning and data processing using the polyGeVero-CT software package (image filtered with the Mean filter: Kernel mode: 2D, Kernel size: 3, Kernel unit: mm, iteration: 1). The following irradiation settings were applied: MLC gap of 5 mm, gantry set at 0°, collimator angles set at 0, 90, 150 and 240°; monitor unit per beam of 1500–7500 MU.

Planes	Planned Angles [°]	Monitor Units			
		1500	2500	5000	7500
		Measured Angles [°]			
Plane 1	0	0.37	359.87	359.80	0.13
Plane 2	90	89.70	90.13	90.22	90.41
Plane 3	150	149.83	149.97	149.70	150.23
Plane 4	240	239.86	240.06	239.81	240.07
		Difference: planned − measured angles [°]			
Plane 1	0	0.37	0.13	0.20	0.13
Plane 2	90	0.30	0.13	0.22	0.41
Plane 3	150	0.17	0.03	0.30	0.23
Plane 4	240	0.14	0.06	0.19	0.07

## Data Availability

The data supporting the reported results are not stored in any publicly archived datasets. The readers can contact the corresponding author for any further clarification of the results obtained.
